# Distinct roles and differential expression levels of Wnt5a mRNA isoforms in colorectal cancer cells

**DOI:** 10.1371/journal.pone.0181034

**Published:** 2017-08-31

**Authors:** Tsui-Chin Huang, Pin-Tse Lee, Ming-Heng Wu, Chi-Chen Huang, Chiung-Yuan Ko, Yi-Chao Lee, Ding-Yen Lin, Ya-Wen Cheng, Kuen-Haur Lee

**Affiliations:** 1 Graduate Institute of Cancer Biology and Drug Discovery, College of Medical Science and Technology, Taipei Medical University, Taipei, Taiwan; 2 Cellular Pathobiology Section, Intramural Research Program, National Institute on Drug Abuse, Rockville, Maryland, United States of America; 3 Graduate Institute of Translational Medicine, College of Medical Science and Technology, Taipei Medical University, Taipei, Taiwan; 4 Graduate Institute of Neural Regenerative Medicine, College of Medical Science and Technology, Taipei Medical University, Taipei, Taiwan; 5 Institute of Bioinformatics and Biosignal Transduction, College of Bioscience and Biotechnology, National Cheng Kung University, Tainan, Taiwan; 6 Cancer Center, Taipei Medical University Hospital, Taipei Medical University, Taipei, Taiwan; National Cancer Center, JAPAN

## Abstract

The canonical Wnt/β-catenin pathway is constitutively activated in more than 90% of colorectal cancer (CRC) cases in which β-catenin contributes to CRC cell growth and survival. In contrast to the Wnt/β-catenin pathway, the non-canonical Wnt pathway can antagonize functions of the canonical Wnt/β-catenin pathway. Wnt5a is a key factor in the non-canonical Wnt pathway, and it plays diverse roles in different types of cancers. It was shown that reintroducing Wnt5a into CRC cells resulted in inhibited cell proliferation and impaired cell motility. However, contradictory results were reported describing increased Wnt5a expression being associated with a poor prognosis of CRC patients. Recently, it was shown that the diverse roles of Wnt5a are due to two distinct roles of Wnt5a isoforms. However, the exact roles and functions of the Wnt5a isoforms in CRC remain largely unclear. The present study for the first time showed the ambiguous role of Wnt5a in CRC was due to the encoding of distinct roles of the various Wnt5a mRNA isoforms. A relatively high expression level of the Wnt5a-short (S) isoform transcript and a low expression level of the Wnt5a-long (L) isoform transcript were detected in CRC cell lines and specimens. In addition, high expression levels of the Wnt5a-S mRNA isoform and low expression levels of the Wnt5a-L mRNA isoform were significantly positively correlated with tumor depth of CRC patients. Furthermore, knockdown of the endogenous expression of the Wnt5a-S mRNA isoform in HCT116 cells drastically inhibited their growth ability by inducing apoptosis through induction of FASLG expression and reduction of TNFRSF11B expression. Moreover, reactivation of methylation inactivation of the Wnt5a-L mRNA isoform by treatment with 5-azacytidine (5-Aza) enhanced the siWnt5a-S isoform's ability to induce apoptosis. Finally, we showed that the simultaneous reactivation of Wnt5a-L mRNA isoform and knockdown of Wnt5a-S mRNA isoform expression enhanced siWnt5a-S isoform-induced apoptosis and siWnt5a-L isoform-regulated suppression of β-catenin expression *in vitro*. High expression levels of the Wnt5a-S mRNA isoform and low expression levels of the Wnt5a-L mRNA isoform were significantly positively correlated with high mRNA levels of β-catenin detection *in vivo*. Altogether, our study showed that, for the first time, different Wnt5a mRNA isoforms play distinct roles in CRC and can be used as novel prognostic markers for CRC in the future.

## Introduction

Aberrant activation of the Wnt/β-catenin signaling pathway is a hallmark of various types of cancer [1]. The canonical Wnt/β-catenin pathway is well-known to be associated with colorectal cancer (CRC) formation. In contrast to the Wnt/β-catenin pathway, the non-canonical Wnt pathway does not signal through β-catenin and can antagonize functions of the canonical Wnt/β-catenin pathway [2, 3]. Wnt5a is a key factor of the non-canonical Wnt pathway and plays diverse roles in different types of cancer. For example, Wnt5a was demonstrated to be upregulated in cancers of the lung, breast, stomach, and prostate [4–7]. Conversely, Wnt5a was shown to inhibit tumor cell proliferation in brain, breast, and thyroid cancers [8–10]. In CRC, the expression level and role of Wnt5a in CRC tumorigenesis are still ambiguous. It was shown that reintroducing Wnt5a into CRC cells, resulted in inhibition of cell proliferation and impairment of cell motility [11]. In addition, downregulation of Wnt5a is associated with a higher tumor grade of CRC patients [12]. However, contradictory results were reported to describe how increased Wnt5a expression is associated with a poor prognosis of CRC patients [13]. Moreover, significant overexpression of Wnt5a was also detected in Skrzypczak *et al*.'s CRC dataset [14] and The Cancer Genome Atlas (TCGA) CRC dataset (https://www.oncomine.org). These results suggest that dysregulation of Wnt5a expression is involved in CRC tumorigenesis, but its exact role is still controversial.

A recent study indicated that the opposing roles for Wnt5a in cancer are due to the encoding of two different splice isoforms [15]. The Wnt5a gene generates two very identical transcripts by utilization of alternative transcription start sites, the corresponding upstream sequences are termed promoters A and B [16], and their products are Wnt5a-long (L) (the Wnt5a-L isoform includes 18 amino acids of the N-terminal domain of the elongated Wnt5a-L isoform) and Wnt5a-short (S) (the Wnt5a-S isoform is the truncated Wnt5a-S form) isoforms, respectively. It was found that the Wnt5a-L isoform inhibits proliferation of breast cancer, cervix cancer, and neuroblastoma cells when ectopically expressed, whereas the ectopic expression of the Wnt5a-S isoform leads to promotion of the proliferation of these cell lines [17]. In CRC, the roles and functions of the Wnt5a isoforms remain largely unclear. Interestingly, in the present study, it was obvious that differential expressions of the Wnt5a isoforms were detected in CRC cells. In addition, distinct roles of the Wnt5a isoforms were examined in CRC cells.

## Materials and methods

### Reagents and antibodies

5-Azacytidine (5-Aza), DMSO, and crystal violet were purchased from Sigma-Aldrich (St. Louis, MO, USA). 5-Aza was dissolved in DMSO at 10 mM as a stock solution and diluted when used. pCMV6-Myc-DDK-tagged-Wnt5a-L isoform plasmid was obtained from Origene (Rockville, MD, USA). Rabbit antibodies against CDK4, poly(ADP-ribose) polymerase (PARP), β-catenin, Cyclin D1, and DNMT1 were obtained from Cell Signaling (Beverly, MA, USA). Rat antibody against pan-Wnt5a was purchased from R&D Systems (Minneapolis, MN, USA) Mouse monoclonal antibodies against caspase-3 and β-actin were respectively purchased from Imgenex (San Diego, CA, USA) and MP Biomedicals (Irvine, CA, USA).

### Tissue samples and ethics statement

CRC tumor tissues were divided into two groups. The first group included 68 tumor samples which were used to analyze mRNA expression of Wnt5a isoforms; the second group included 123 tumor samples which were used to analyze protein expression of Wnt5a-S isoform. Both groups underwent surgical resection for CRC in the Department of Surgery, Taipei Medical University Hospital (Taipei, Taiwan). Informed written consent was obtained from all patients and/or guardians for the use of their resected specimens. Acquisition of samples and their subsequent examination were approved by the Institutional Review Board (IRB) of Taipei Medical University (TMU-JIRB No.: 201312039). None of the participants had a previous history of cancer. The characteristics of the clinical specimens of CRC used in the study were summarized in Table 1 and S1 Table.

**Table 1:** Association of Wnt-5a mRNA isoforms expression and clinical parameters in tumor tissues of colorectal cancer patients.

Wnt5a			
Clinicopathological features	S isoform High & L isoform Low (n = 41)	L isoform High & S isoform Low (n = 27)	*p* value
Gender			
Female	23	11	
Male	18	16	0.215
T factor			
1	0	1	
2	4	7	
3	22	12	
4	15	17	0.172
T factor			
1+2	16	13	
3+4	31	63	0.037
N factor			
0	13	11	
1+2	28	16	0.745
M factor			
0	36	23	
1	5	4	0.103
TNM Stage			
I	1	5	
II	10	8	
III	22	11	
IV	8	13	0.103

### Cell lines and cell culture

CRC cell lines were provided by Prof. YW Cheng and Prof. H Lee’s laboratory. All CRC cell lines were cultured in RPMI-1640 (Gibco, Grand Island, NY, USA), supplemented with 10% fetal bovine serum (FBS) (Gibco, Grand Island, NY, USA) and antibiotics. CRL-1459/CCD-18Co cells (non-malignant human colon cells) were provided by Prof. PJ Lu's laboratory and cultured in minimum essential Eagle's medium (Gibco, Grand Island, NY, USA), supplemented with 10% FBS and antibiotics. All cell lines used in this study were summarized in S2 Table.

### Cell transfection and cell viability assay

Cells were transfected with control small interfering (si)RNA, the siWnt5a-L isoform, or the siWnt5a-S isoform using Lipofectamine (Invitrogen, Paisley, UK) according to the manufacturer's instructions. siRNA sequences of the different Wnt5a isoforms were obtained from Bauer *et al*. [17] and synthesized by Purigo Biotech (Taipei, Taiwan). Cell viability was determined by the crystal violet staining method, as described previously [18]. In brief, cells were plated in 96-well dishes at a density of 4000 cells/ml with or without treatment, and viable cells were stained with 0.5% crystal violet in 30% ethanol for 10 min at room temperature for the indicated time. Plates were then washed four times with tap water. After drying, cells were lysed with a 0.1 M sodium citrate solution, and dye uptake was measured at 550 nm using a 96-well plate reader. Cell viability was calculated from the relative dye intensity compared to untreated samples.

### Focus formation assays

HCT116 cells were seeded at 1.5 × 10^5^ cells in 35-mm culture dishes and allowed to adhere overnight before transfection with control siRNA, the siWnt5a-L isoform, or the siWnt5a-S isoform. After 24 h, cells were trypsinized and seeded at 5000 cells in 6-well culture dishes for 11 days, then fixed with methanol, and stained with 0.5% crystal violet. Foci of greater than 5 mm in size were manually counted. All transfections were repeated three times, and average focus counts and standard deviations (SDs) were calculated.

### Formation of spheroids

After 24 h, cells were transfected with control siRNA, the siWnt5a-L isoform, or the siWnt5a-S isoform, and 200-μl cell suspensions with 5 × 10^3^ cells were seeded into each well of a 96-well NanoCulture^®^ plate (SCIVAX USA, Woburn, MA, USA). The plates were incubated at 37°C in 5% CO_2_. On day 3, loose spheroids had formed, and 100 μl/well of medium was replaced with fresh medium. After another 3 days of culture, larger and tighter 6-day-old spheroids had formed, and images were captured by light microscopy.

### Apoptosis polymerase chain reaction (PCR) array and quantitative reverse-transcription (RT)-(q)PCR

Total RNA was extracted from control siRNA and siWnt5a-S isoform-transfected HCT116 cells using a Qiagen RNeasy kit (Valencia, CA, USA) and Qiashredder columns according to the manufacturer’s instructions (Qiagen). One microgram of total RNA was reverse-transcribed to complementary (c)DNA using a Reaction Ready^™^ First Strand cDNA Synthesis Kit (SABiosciences, Frederick, MD, USA) and applied to the Apoptosis PCR Array following SABiosciences' RT-PCR manual (cat. no. PAHS-012Z, 96-well format). Plates were processed in an Applied Biosystems StepOnePlus^™^ Real-Time PCR System (Foster City, CA, USA) using an automated baseline and threshold cycle detection. Data were interpreted using SABiosciences’ web-based PCR array analytical tool. To detect expression levels of regulated genes, the Wnt5a-L and -S isoforms, and GAPDH, specific products were amplified and detected using the cycle profile of the Qiagen miScript SYBR green PCR starter kit. The relative gene expression level was calculated by comparing the cycle times for each target PCR. The mRNA expression of β-catenin in CRC tissues were divided into two groups: low expression (<Mean value) and high expression (>Mean value). Sequences of primers used in this study are listed as follows: Wnt5a-L isoform: forward 5′-CCGGTCGCTCCGCTCGGAT-3′ and reverse 5′-GCATGTGGTCCTGATACAAGT-3′; Wnt5a-S isoform: forward 5′-CGCCTCCTTGGCAGCCTCT-3′ and reverse 5′-GCATGTGGTCCTGATACAAGT-3′; FASLG: forward 5′-CCAGCCAGATGCACACAGC-3′ and reverse 5′- CATTCCAGAGGCATGGACC-3′; TNFRSF9: forward 5′-GCTGACGTCGACTGCGTTG-3′ and reverse 5′-ATCGGCAGCTACAGCCATC-3′; TNFRSF11B: forward 5′-CCTGTGTGAGGAGGCATTC-3′ and reverse 5′-CTTGTGAGCTGTGTTGCCG-3′; β-catenin: forward 5′-TGCTAAATGACGAGGACCAG-3′ and reverse 5′-TGAGGAGAACGCATGATAGC-3′; and GAPDH: forward 5′-AATCCCATCACCATCTTCCA-3′ and reverse 5′-TGAGTACGTCGTGGAGTCCA-3′.

### Western blot analysis

Cell lines were placed in lysis buffer at 4°C for 1 h. Protein samples were electrophoresed using 8%~15% sodium dodecylsulfate (SDS)-polyacrylamide gel electrophoresis (PAGE) and performed as previously described [19].

### Immunohistochemical (IHC) analysis

The tissue sections were stained with a standard IHC protocol. Briefly, slides were processed by deparaffinization, antigen retrieval, and -blocking. After blocking, the slides were incubated with a primary antibody against pan-Wnt5a, followed by a biotin-conjugated secondary antibody. The intensity of staining was scored as follows: 0 point, negative; 1 point, weakly positive; 2 points, moderately positive; or 3 points, strongly positive. The percentage of positive tumor cells (0–100%) was multiplied by the intensity of all proteins staining, and the overall score ranged from 0 to 300. The proteins expression scores (0–300) were divided into two groups: low expression (0–150) and high expression (151–300).

### Apoptosis assays

Apoptosis assays were conducted using a flow cytometry-based approach. In order to evaluate the effects of different Wnt5a mRNA isoforms to induce apoptosis, HCT116 cells (2.5 × 10^5^) were transfected with different siWnt5a isoforms for 48 h, and then cells were collected in culture medium, mixed with the Muse annexin V and Dead Cell Reagent, and analyzed using a Muse Cell Analyzer (EMD Millipore, Billerica, MA, USA).

### Statistical analyses

All analyses were conducted with at least three independent experiments. Results are presented as the mean±standard deviations (SDs). We used one-tailed Student's *t*-tests for all comparison experiments. A Chi-squared test was used to compare Wnt5a mRNA isoforms expression with clinicopathological parameters and compare mRNA expression levels of the Wnt5a-L and -S isoforms with the β-catenin mRNA expression level. All statistical analyses were performed using GraphPad Prism software (GraphPad, San Diego, CA, USA).

## Results

### Expression of Wnt5a mRNA isoforms in CRC cells

**Fig 1:**
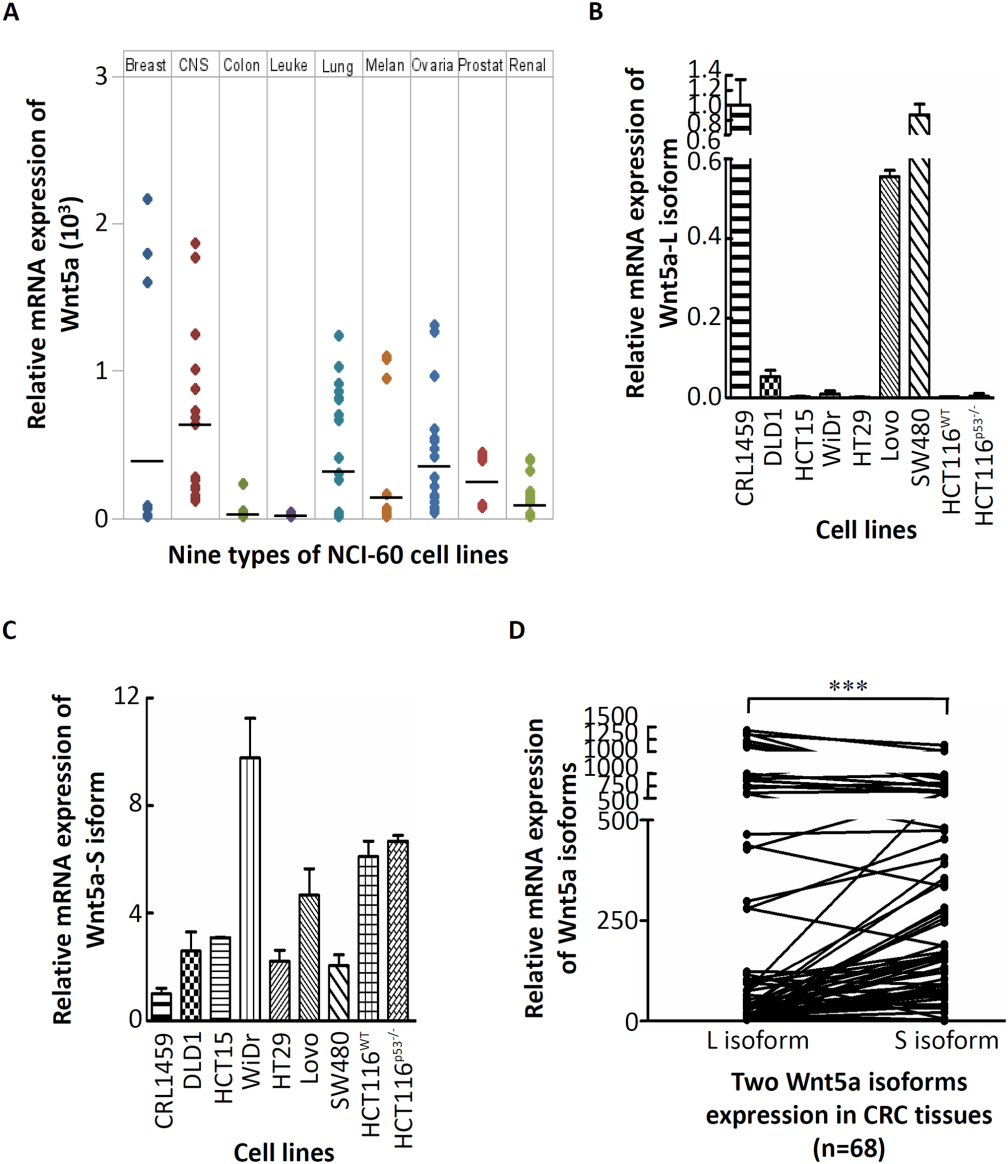
Wnt5a mRNA isoforms expressions in colorectal cancer (CRC) cells. (A) Expression levels of Wnt5a in nine types of NCI60 cancer cell lines which were retrieved from the CellMiner database. The Wnt5a-long (L) isoform (B) and—short (S) isoform (C) mRNA analyses were conducted on RNA isolated from nine CRC cell lines and one non-cancerous human CRL-1459 colon cell line. (D) The Wnt5a-L isoform and -S isoform mRNA expression levels were detected in CRC tissues.

To understand the expression correlation between different Wnt5a mRNA isoforms and CRC cells, we first investigated mRNA expression levels of Wnt5a in nine NCI-60 cell lines. As shown in Fig 1A, among the nine cancer cell lines, expression levels of Wnt5a mRNA were quite varied. In addition, we found that low expression of Wnt5a mRNA was detected in CRC cell lines. However, the expression level of Wnt5a is still controversial in CRC [13, 20]. To understand the expression of different Wnt5a mRNA isoforms in CRC, we first investigated mRNA expression levels of Wnt5a isoforms in a panel of CRC cell lines by an RT-qPCR analysis. Results showed that high mRNA expression of the Wnt5a-L isoform was detected in non-cancerous human CRL-1459 colon cells. As expected, relatively low mRNA expression of the Wnt5a-L isoform was detected in most CRC cell lines compared to that in CRL-1459 cells (Fig 1B). In addition, it was demonstrated that the Wnt5a-L isoform is weakly methylated in the Lovo cell line, whereas it is unmethylated in SW480 cells with strong expression [20]. This result is consistent with our findings which showed that high mRNA expression of the Wnt5a-L isoform was detected in the Lovo and SW480 cell lines (Fig 1B). In contract, a relatively high mRNA expression level of the Wnt5a-S isoform was detected in most CRC cell lines compared to that in CRL-1459 cells (Fig 1C). Next, transcript levels of Wnt5a isoforms in CRC tissues were further investigated in the first group of CRC tumor tissues (n = 68). As shown in Fig 1D, 60.3% of CRC tissues had a relatively high expression level of the Wnt5a-S mRNA isoform and a low expression level of the Wnt5a-L mRNA isoform (*p*<0.001). Further, the association of Wnt5a mRNA isoforms expression level and several clinicopathologic factors of CRC patients were analyzed. As shown in Table 1, tumor tissues with high expression levels of the Wnt5a-S mRNA isoform and low expression levels of the Wnt5a-L mRNA isoform were significantly positively correlated with tumor depth of CRC patients. To determine the level of Wnt5a isoforms protein expression in CRC cell lines, the expression of Wnt5a protein isoforms were detected by using pan-Wnt5a antibody. Compared with CRL-1459, high expression of pan-Wnt5a protein was detected in all CRC cell lines except Lovo (S1 Fig). In addition, the protein expression levels of Wnt5a isoforms were determined by IHC in the second group of CRC tumor tissues (n = 123). As shown in S1 Table, we found that pan-Wnt5a protein was highly expressed in over 60% of CRC tumor tissues. In addition, statistically significant relationship was found between pan-Wnt5a protein and tumor depth (*p* = 0.031), tumor metastasis (*p* = 0.011), and clinical staging CRC tumor tissues (*p* = 0.001). Taken together, although upregulation of pan-Wnt5a protein was detected in CRC cell lines and tissues, but, the exact association between expression status of Wnt5a isoforms and CRC only can determine by analyzed Wnt5a mRNA isoforms.

### Growth regulation of different Wnt5a mRNA isoforms in CRC cells

**Fig 2:**
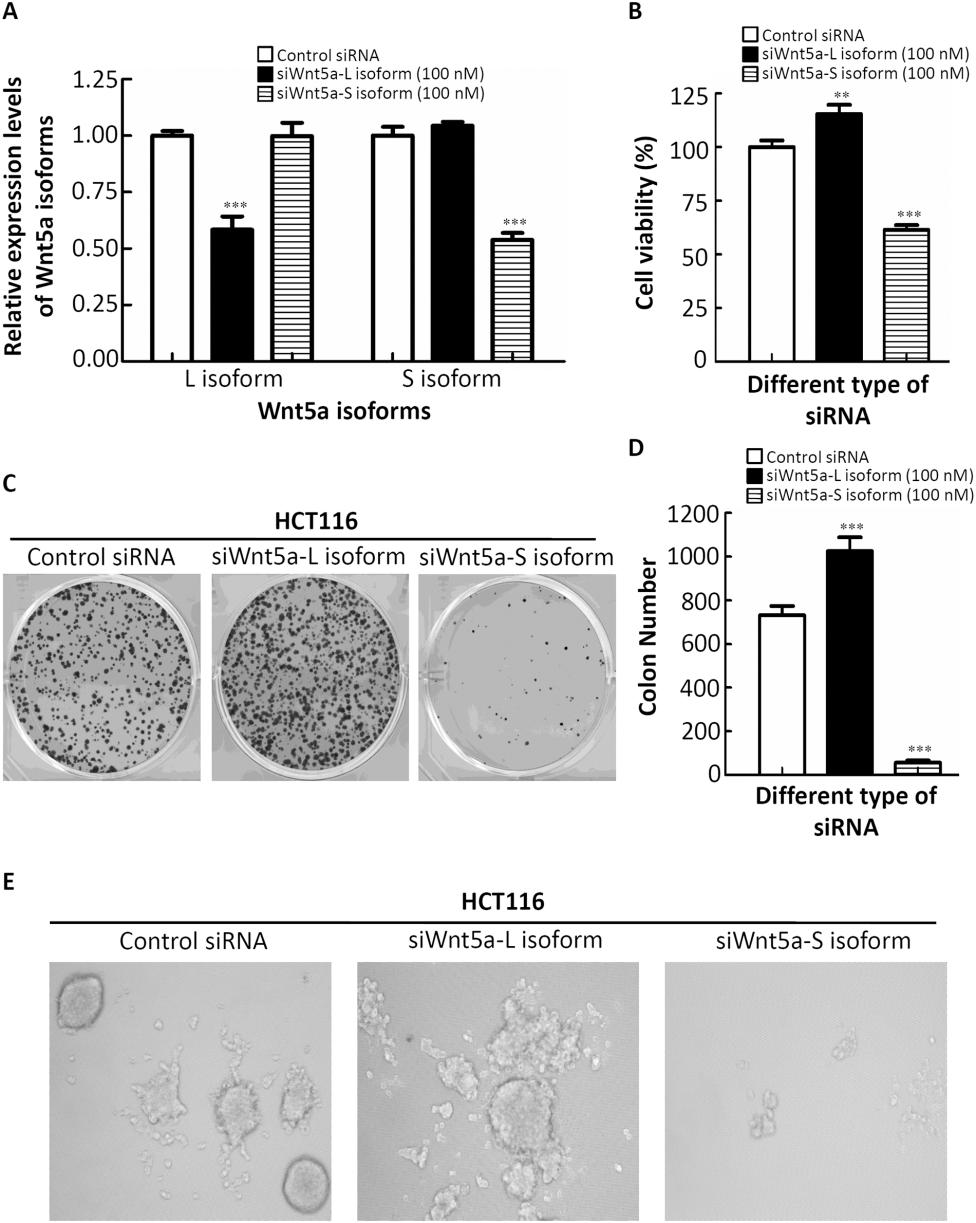
Effects of the Wnt5a-long (L) and Wnt5a-short (S) mRNA isoforms on cell proliferation and spheroid formation of colorectal cancer (CRC) cells. (A) Validation of expression levels of the different Wnt5a mRNA isoforms after transfection with specific siRNAs of the different Wnt5a isoforms for 48 h in the HCT116 cell line. (B) The proliferative ability of HCT116 cells after transfection with specific siRNAs of different Wnt5a isoforms. (C) HCT116 cells were transfected with specific siRNAs of different Wnt5a isoforms, and foci were visualized after crystal violet staining. (D) The combined results of all transfections, wherein total focal counts ± SD are illustrated. (E) HCT116 cells were grown in 3D NanoCulture Plates (NCPs) with transfection with specific siRNAs of different Wnt5a isoforms. In the 3D-NCP condition, siWnt5a-L isoform-transfected spheroid cells appeared to increase in spheroid size, but siWnt5a-S isoform-transfected spheroid cells appeared to spread out from the spheroids, and the spheroid sizes were reduced.

Next, to further confirm the functions of different Wnt5a mRNA isoforms in CRC cells, specific siRNAs of the different Wnt5a mRNA isoforms were designed from Bauer *et al*. [17]. As shown in Fig 2A, siRNAs were able to specifically knock down up to 50% of their targets as determined by an RT-qPCR. Importantly, each Wnt5a isoform was selectively inhibited by the corresponding siRNA without affecting levels of the other isoform (Fig 2A). Furthermore, knockdown of the Wnt5a-L mRNA isoform increased cell proliferation in HCT116 cells as detected by an MTT assay (Fig 2B). In contrast, cell growth was inhibited by knockdown of the Wnt5a-S mRNA isoform in HCT116 cells (Fig 2B). To investigate the effects of different Wnt5a mRNA isoforms on the tumorigenic phenotypes of CRC cells, we examined the effects of different Wnt5a mRNA isoforms on colony formation in a focus-forming assay. Strikingly, we found significantly increased colony numbers and sizes in HCT116 cells with knockdown of the endogenous expression of the Wnt5a-L mRNA isoform compared to control siRNA (*p*<0.001) (middle panel of Fig 2C and 2D). In contrast, knockdown of the endogenous expression of the Wnt5a-S mRNA isoform in HCT116 cells drastically inhibited their growth ability, as shown by decreased colony numbers and sizes (*p*<0.001) (right panel of Fig 2C and 2D). Three-dimensional (3D) multicellular spheroid culture conditions closely mimic the *in vivo* cell environment and have been used to demonstrate activation of transcription programs that lead to tumor survival and drug resistance [21]. Next, we sought to determine the effect of administering different Wnt5a mRNA isoforms on colon carcinoma multicellular spheroid cultured cells *in vitro*. In 3D culture, HCT116 cells showed a disorganized structure with poor cell-cell contacts (Fig 2E, left panel). Reduced Wnt5a-L mRNA isoform expression in 3D cultures of HCT116 cells formed larger colonies compared to the control siRNA counterpart (Fig 2E, middle panel). In contrast, cells with reduced Wnt5a-S mRNA isoform levels formed smaller colonies (Fig 2E, right panel). Taken together, these observations indicate that each mRNA isoform of Wnt5a has distinct biochemical properties in regulating CRC cell growth.

### Molecular function of different Wnt5a mRNA isoforms in CRC cells

**Fig 3:**
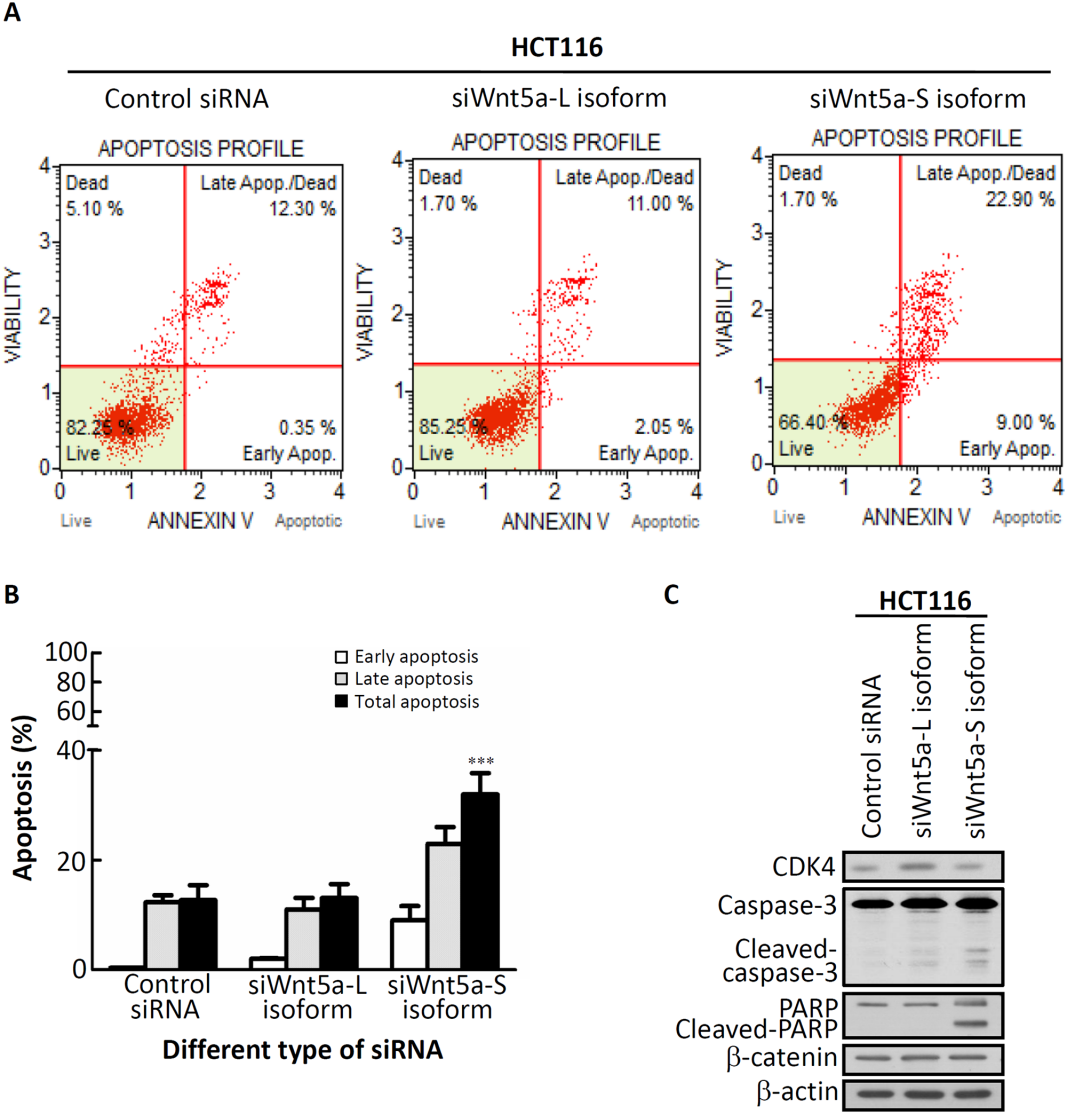
siWnt5a-short (S) isoform-induced apoptotic death in HCT116 cells. (A) HCT116 cells were transfected with specific siRNAs of different Wnt5a isoforms for 48 h. Cells were subjected to an annexin-V assay using a Muse cell analyzer. The method led to four different populations of cells: live cells (annexin-V negative (lower right quadrant)), early apoptotic cells (positive for annexin-V (lower right corner)), late apoptotic/dead cells (annexin V positive (upper right quadrant)), and non-apoptotic dead cells (upper left quadrant). (B) Quantification of total apoptotic cells (early apoptosis + late apoptotic cells) determined by annexin-V positivity. (C) HCT116 cells transfected with specific siRNAs of different Wnt5a isoforms for 48 h, subjected to a Western blot analysis, and probed for CDK4, caspase-3, poly(ADP ribose) polymerase (PARP), and β-catenin antibodies, respectively. β-Actin was used as the loading control.

We then examined whether knockdown of Wnt5a-S mRNA isoform-induced cytotoxicity is mediated by a cell cycle effect or apoptotic processes, using propidium iodide (PI) staining, annexin V staining, and antibodies against a cell cycle marker and apoptosis markers. Results of the cell cycle distribution revealed that transfection with the Wnt5a-S mRNA isoform caused a decrease in the G_0_/G_1_ phase and accumulation of the G_2_/M phase compared to transfection with control siRNA (Table 2). HCT116 cell apoptosis was assessed by annexin V staining. As shown in Fig 3A and 3B, transfection with Wnt5a-S mRNA isoform siRNA for 48 h induced a significant increase in HCT116 cell apoptosis (31.9% ± 3.90%) compared to control siRNA (*p*<0.001). Results of the Western blot analysis revealed that Wnt5a-L mRNA isoform knockdown increased cell proliferation as assessed by CDK4 accumulation (Fig 3C). In addition, transfection with Wnt5a-S mRNA isoform siRNA caused cleavage of caspase-3 and PARP (Fig 3C). However, there was no significant change in the expression level of β-catenin in Wnt5a-L or Wnt5a-S isoform siRNA-transfected HCT116 cells. Next, to further investigate whether overexpression of Wnt5a isoforms could execute its molecular function, we performed the gain of function experiment by introducing Wnt5a-L isoform-expressing plasmid (pCMV6-Myc-DDK-tagged-Wnt5a-L isoform) or empty vector in HCT116 cells, followed by cell viability, Western blotting for cell cycle regulators and apoptosis marker examinations. As shown in Figure A in S2 Fig, cell viability assay revealed that significantly reduced cell viability in pCMV6-Myc-DDK-tagged-Wnt5a-L isoform-transfected HCT116 cells in the dose dependent manner compared to cells transfected with pCMV6 vector. Moreover, Western blot results revealed that overexpression of Wnt5a-L isoform protein was detected by using Myc-tag antibody (Figure B in S2 Fig). In addition, reduction of cyclin D1 and CDK4 protein expression as well as induction PARP cleavage were observed in pCMV6-Myc-DDK-tagged-Wnt5a-L-transfected HCT116 cells (Figure B in S2 Fig). Overall, these results show that different Wnt5a isoforms exert distinct activities in CRC cells.

**Table 2:** Cell-cycle distribution of HCT116 cells transfected with different siWnt5a isoforms.

Cell-cycle phase	DNA contents (%)		
	Control siRNA	siWnt5a-long isoform	siWnt5a-short isoform
G_0_/G_1_	46.1±2.34	48.0±2.54	42.9±2.14
S	20.6±1.56	20.3±1.46	20.2±1.42
G_2_/M	24.9±1.87	25.2±1.76	28.3±1.59

### Aberrant expression of Wnt5a-S mRNA isoform-mediated regulation of two apoptosis-related genes in colorectal tumors is a strong predictor of a poor prognosis

**Fig 4:**
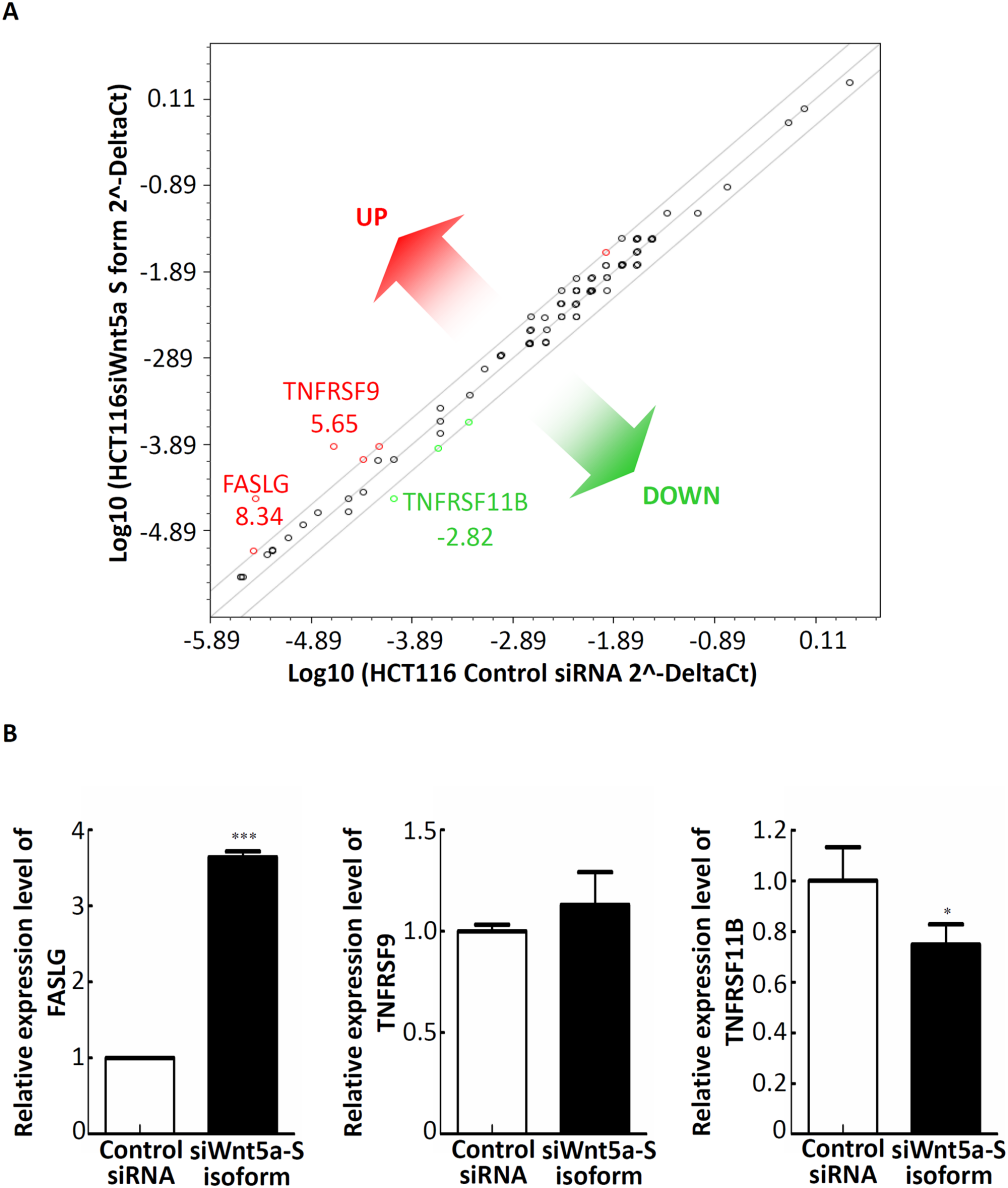
Wnt5a-short (S) mRNA isoform-mediated regulation of the expressions of multiple genes associated with apoptosis. (A) Total RNA from control siRNA and siWnt5a-S isoform-transfected HCt116 cells was characterized using a Human Apoptosis PCR Array. The figure shows a scatter plot of differences in relative transcript abundances of 84 key genes that either change their expression during apoptosis or regulate those apoptosis-related gene expression changes. Three genes showed significant changes in siWnt5a-S isoform-transfected HCT116 cells compared to control siRNA-transfected cells. (B) Dysregulated genes identified by the Apoptosis PCR Array experiments and verified by an independent RT-qPCR of HCT116 cells. An RT-qPCR was performed using independent primers to those used in the array experiments. Relative mRNA abundances were calculated using GAPDH as an endogenous control. RT-qPCR values are the mean ± SD of *n* = 3 and normalized to the mean of control siRNA-transfected cells. * *p*<0.05, *** *p*<0.001.

**Fig 5:**
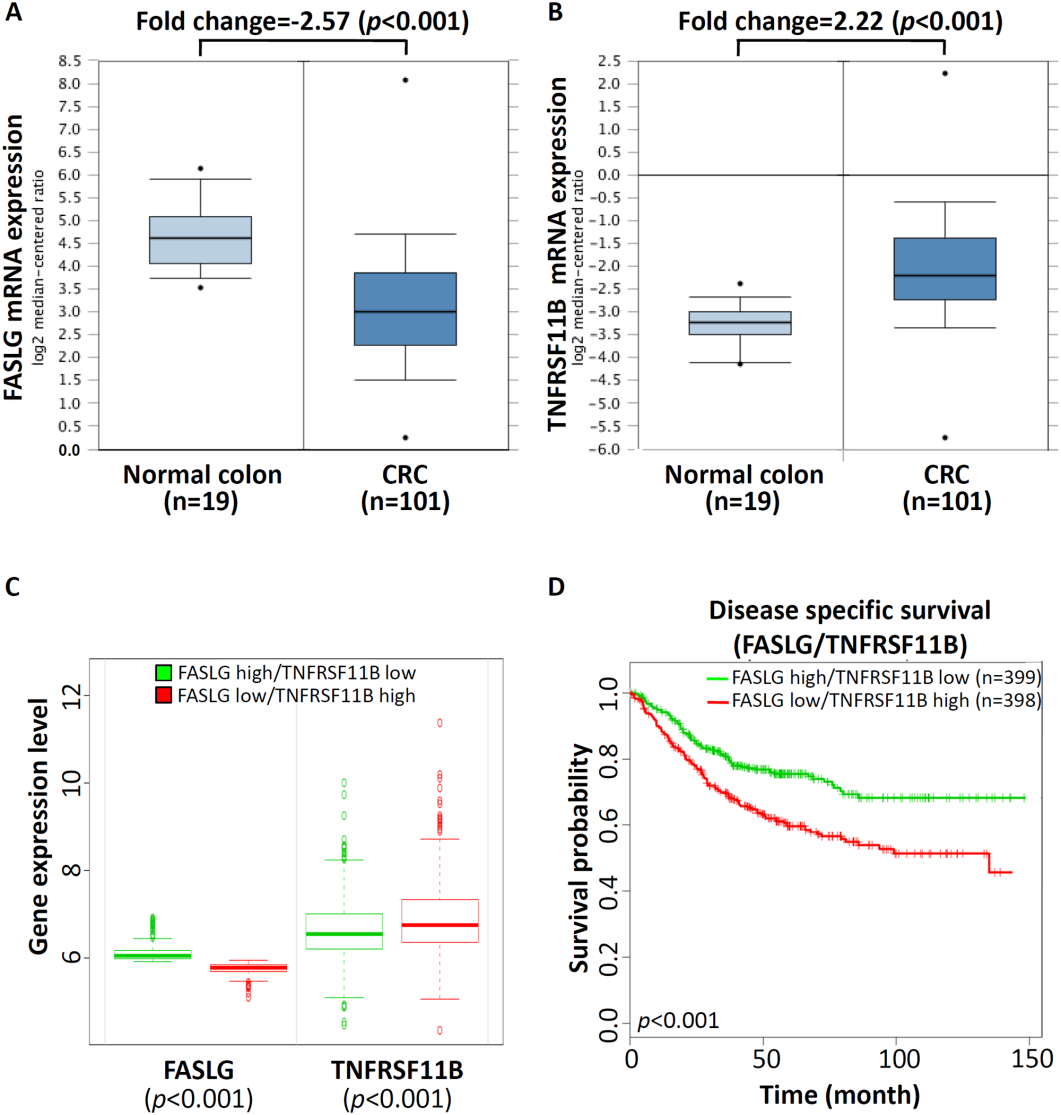
Aberrant expressions of Wnt5a-short (S) mRNA isoform-modulated apoptosis-related genes associated with the survival rate of colorectal cancer (CRC) patients. mRNA expressions of FASLG (A) and TNFRSF11B (B) in normal colorectal and colorectal tumor tissues were obtained from the Oncomine database (http://www.oncomine.org/). (C) Box plots comparing differences in FASLG and TNFRSF11B mRNA expressions between risk groups using a *t*-test. (D) Two-gene (FASLG and TNFRSF11B) combinations were used to accurately predict patient outcomes using Kaplan-Meier analyses from the CRC Survival Metabase showing stratification of FASLG and TNFRSF11B for longer-surviving (patients with high expression of FASLG and low expression of TNFRSF11B) versus shorter-surviving patients (patients with low expression of FASLG and high expression of TNFRSF11B).

Above results showed that Wnt5a-S mRNA isoform was overexpressed in CRC cell lines and tissues at mRNA levels. In addition, knockdown of the Wnt5a-S mRNA isoform causes growth inhibition of CRC cells by inducing apoptosis. Thus, to understand the role of Wnt5a-S mRNA isoform-mediated regulation of apoptosis of CRC cells, we measured expression levels of a set of apoptosis-relevant genes after transfection of control siRNA or the siWnt5a-S isoform in HCT116 cells using RT^2^ Profiler PCR Arrays. A three-fold or greater difference in mRNA expression levels was used as the cutoff to determine significant regulatory effects on genes involved in apoptosis. Of the 84 apoptotic genes tested, three genes showed significant changes in siWnt5a-S isoform-transfected HCT116 cells compared to control siRNA-transfected cells (Fig 4A). We further adopted a real-time qPCR to confirm expressions of some selected genes (*FASLG*, *TNFRSF9*, and *TNFRSF11B*) after transfection of cells with 100 nM of the siWnt5a-S isoform. Significant increases in the mRNA expression level of the *FASLG* gene and a significant decrease in the mRNA expression level of the *TNFRSF11B* gene were found after transfecting cells with 100 nM of the siWnt5a-S isoform (Fig 4B). Further, to understand the role of Wnt5a-S mRNA isoform-mediated regulation of apoptosis-related genes in CRC tissues, we analyzed *FASLG* and *TNFRSF11B* mRNA expression profiles using existing complementary (c)DNA microarray datasets deposited in the Oncomine database. In TCGA microarray dataset of the Oncomine website with colorectal tumor and normal colorectal tissues (https://www.oncomine.org/resource/ui/component/dataset.html?component=d:156636494), a significant decrease in the mRNA expression of FASLG (Fig 5A) and a significant increase in the mRNA expression of TNFRSF11B (Fig 5B) were found in colorectal tumor tissues compared to normal colorectal tissues. To evaluate the prognostic significance of mRNA expressions of FASLG and TNFRSF11B, we analyzed FASLG and TNFRSF11B expression levels of CRC with outcomes available in the CRC Survival Metabase [22]. As shown in Fig 5C, expression levels of FASLG and TNFRSF11B could be used to stratify patients by K-means clustering into two subgroups (low- and high-risk groups) which exhibited significant differences (*p*<0.001) in CRC-specific survival according to a Kaplan-Meier survival analysis (Fig 5D). These results indicated that upregulation of the Wnt5a-S isoform caused downregulation of FASLG and upregulation of TNFRSF11B in CRC, and their aberrant expressions were inversely correlated with a CRC patient's prognosis.

### Simultaneous activation of the Wnt5a-L mRNA isoform and knockdown of the Wnt5a-S mRNA isoform enhance apoptosis, and their aberrant expressions were associated with β-catenin expression

**Fig 6:**
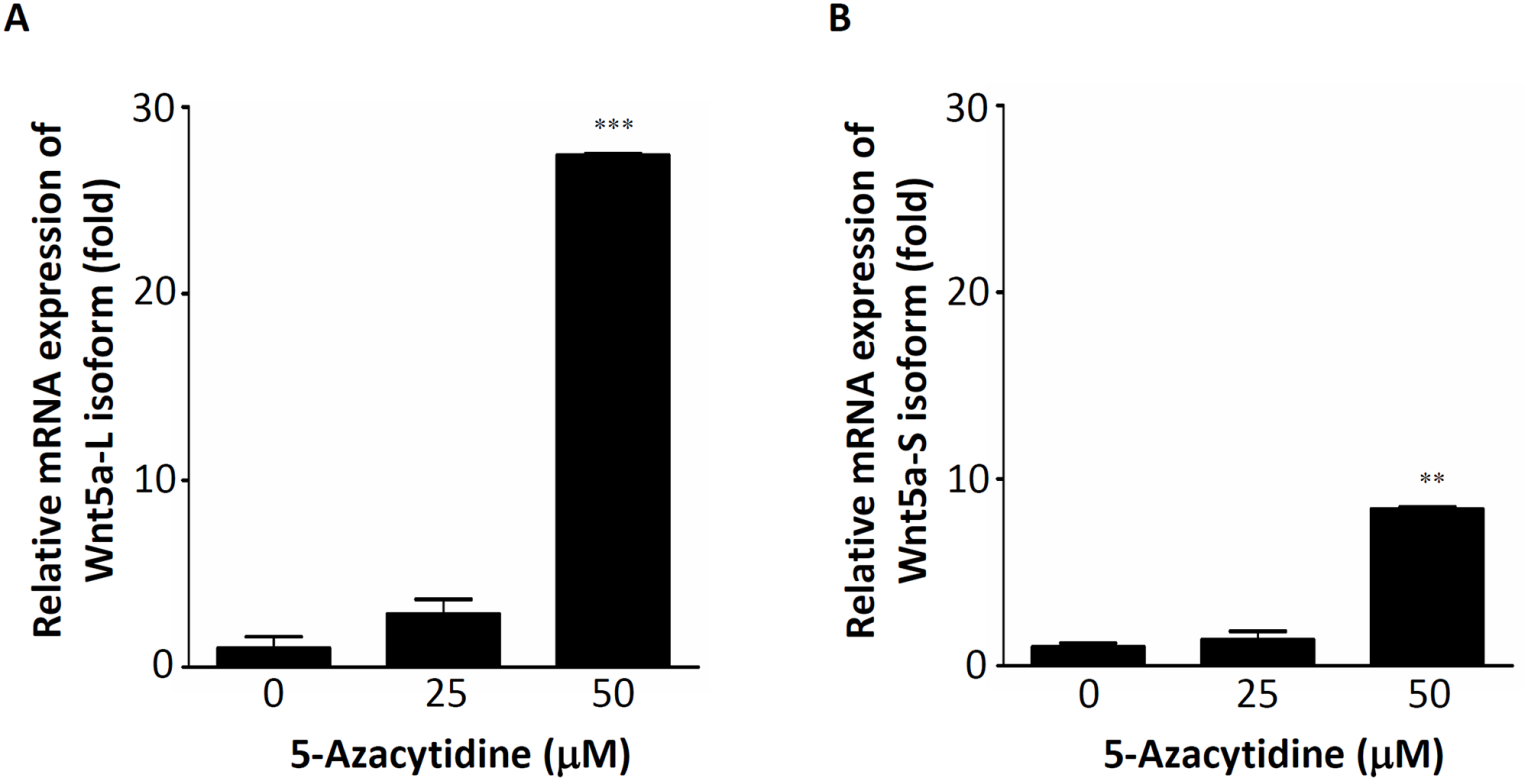
Re-activation of Wnt5a mRNA isoforms by treatment with a demethylation agent. Re-activation of the Wnt5a-long (L) mRNA isoform **(A)** or Wnt5a-short (S) mRNA isoform **(B)** after treatment with 5-azacytidine (5-Aza) at the indicated concentration.

**Fig 7:**
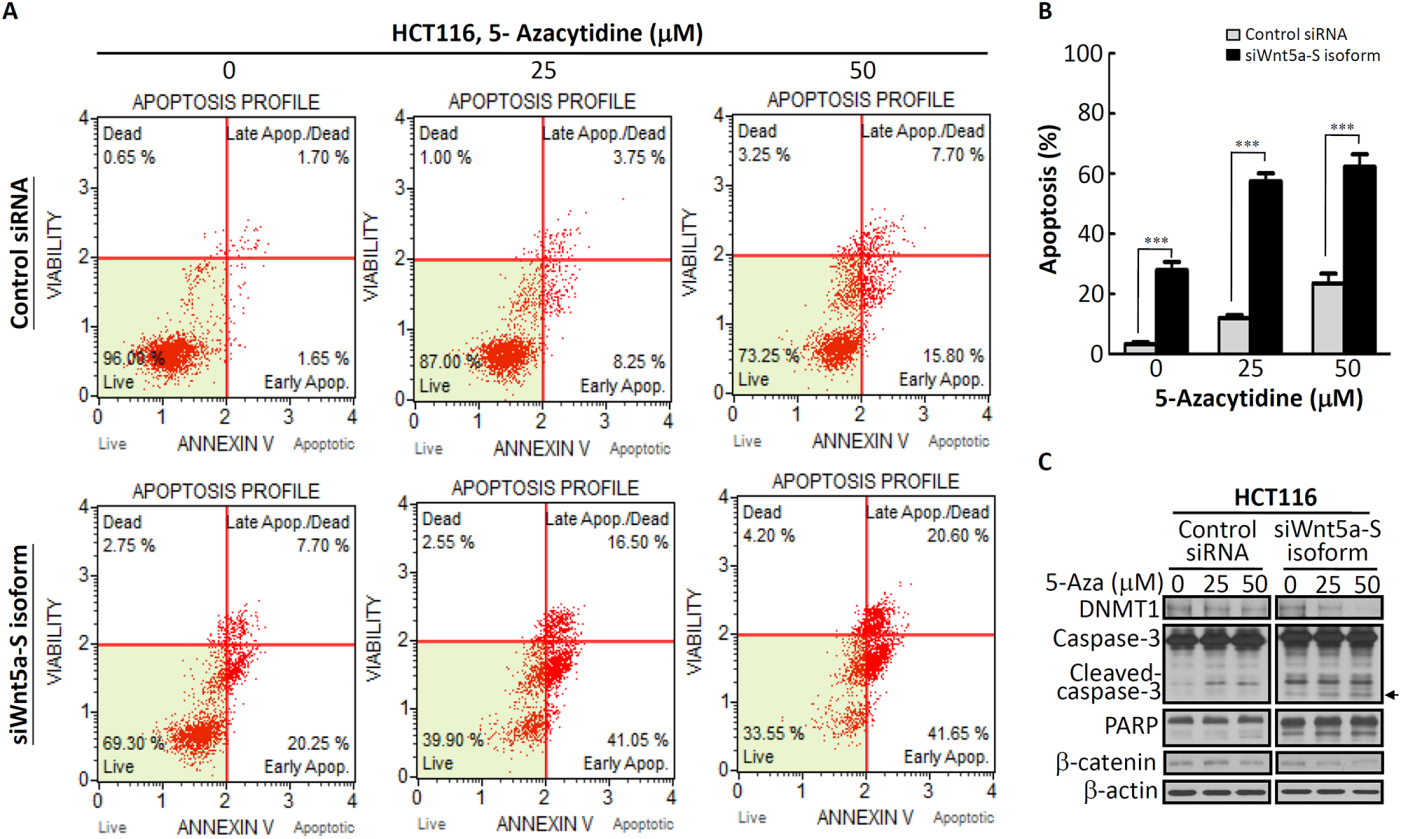
Knockdown of the Wnt5a-short (S) mRNA isoform enhanced the sensitivity of colorectal cancer (CRC) cells to 5-azacytidine (5-Aza). (A) The Wnt5a-S mRNA isoform was silenced for 48 h with 100 nM of specific siRNA, following which cells were treated with 5-Aza at the indicated concentrations for an additional 48 h. Cells were stained with annexin V and analyzed using a Muse Cell Analyzer. (B) The quantification result of annexin V-positive cells with knockdown of the Wnt5a-S isoform alone or in the presence of 5-Aza. (C) Western blot analysis of DNMT1, caspase-3, poly (ADP ribose) polymerase (PARP) cleavage, and β-catenin in HCT116 cells transfected with control siRNA or Wnt5a-S isoform-specific siRNA oligomers for 48 h and subsequently incubated with 5-Aza at the indicated concentrations for an additional 48 h. Arrow symbol indicated that siWnt5a-S isoform knockdown HCT116 cells in the present with 5-Aza showed more significant at inducing caspase-3 cleavage than control siRNA-transfected HCT116 cells in the presence of 5-Aza. β-actin is shown as the loading control.

The Wnt5a gene was split into five conserved exons, and transcription initiation sites of the Wnt5a-L and Wnt5a-S isoforms located in exon 1 and exon 2 were found to be respectively driven by promoters A and B [16]. In addition, the Wnt5a promoter contains a typical CpG island spanning the core promoter, exon 1, and part of intron 1, the frequent methylation of which is detected in CRC tumors (48%), but only occasionally in paired normal colon tissues [20]. Moreover, a schematic structure of the sequence of the Wnt5a promoter showed that a hypermethylation region of the Wnt5a promoter was very close to promoter A [16, 20, 23]. Thus, we suspect that downregulation of the Wnt5a-L mRNA isoform in CRC cells might be mediated by regulation of DNA hypermethylation. DNA hypermethylation can be reversed by DNA-demethylating agents. 5-Aza is a demethylating agent which can restore the expression of genes silenced by DNA methylation. Therefore, HCT116 cells were treated with different concentrations of 5-Aza, and expressions of different Wnt5a mRNA isoforms were analyzed by an RT-qPCR analysis. As shown in Fig 6A, about 27-fold induction of the Wnt5a-L mRNA isoform was detected after 50 μM 5-Aza treatment. Moreover, about 8-fold induction of the Wnt5a-S mRNA isoform was also observed after 50 μM 5-Aza treatment (Fig 6B). To understand the effect of 5-Aza on pan-Wnt5a isoforms protein expression level, the expression of pan-Wnt5a isoforms protein were detected after different concentration of 5-Aza-treated HCT116 cells. As shown in S3 Fig, expression of pan-Wnt5a isoforms protein were slightly changed in different concentration of 5-Aza-treated HCT116 cells. These results indicated that the Wnt5a-L mRNA isoform can be significantly reactivated by 5-Aza treatment. The above results (Figs 1, 2, and S2) indicate that the Wnt5a-L mRNA isoform plays a tumor-suppressor role in CRC. In addition, we found that the Wnt5a-L mRNA isoform can be reactivated by 5-Aza treatment. Thus, reactivation of the Wnt5a-L mRNA isoform by 5-Aza treatment was then hypothesized to enhance cell apoptosis in siWnt5a-S isoform-knockdown HCT116 cells. To test this hypothesis, HT116 cells were transfected with control siRNA or the siWnt5a-S isoform, followed by 5-Aza treatment at the indicated concentration and analysis by annexin V staining. As shown in Fig 7A, siWnt5a-S isoform-knockdown HCT116 cells showed significantly increased cell apoptosis by ~28% compared to the control siRNA group (Fig 7A, left panel). In addition, the siWnt5a-S isoform-knockdown group showed severe and significantly increased cell apoptosis by ~57% in HCT116 cells treated with 25 μM 5-Aza (Fig 7A, middle panel) or severe and significantly increased cell apoptosis by ~62% in HCT116 cells treated with 50 μM 5-Aza compared to the control siRNA group (Fig 7A, right panel). Fig 7B quantifies the total percent of apoptotic cells as determined by an annexin V assay. As one hallmark of apoptosis is activation of cell death machinery caspases, which are activated in response to both intrinsic and extrinsic cues [24], we next examined caspase-3 activity by subjecting whole-cell lysates of siWnt5a-S isoform-transfected HCT116 cells with or without 5-Aza in a Western blot analysis. Western blotting results revealed that 5-Aza caused downregulation of DNMT1 (Fig 7C). Furthermore, siWnt5a-S isoform knockdown was more significant at inducing caspase-3 and PARP cleavage in 5-Aza-treated HCT116 cells than in control siRNA-transfected 5-Aza-treated HCT116 cells (Fig 7C). Moreover, downregulation of β-catenin was detected in siWnt5a-S isoform-transfected HCT116 cells in the presence of 50 μM 5-Aza (Fig 7C). Furthermore, correlations of Wnt5a isoforms and β-catenin expression in tumor tissues of CRC patients were analyzed. As shown in Table 3, tumor tissues with high expression levels of the Wnt5a-S isoform and low expression levels of the Wnt5a-L isoform were significantly positively correlated with high levels of β-catenin. Collectively, these results showed that reactivation of the Wnt5a-L mRNA isoform by treatment with 5-Aza can enhance the siWnt5a-S isoform to induce apoptosis in HCT116 cells. In addition, these results also show that simultaneous upregulation of the Wnt5a-S mRNA isoform and downregulation of the Wnt5a-L mRNA isoform can cause high expression of β-catenin in CRC cells.

**Table 3:** Correlations of Wnt5a mRNA isoforms and β-catenin transcripts expression level in colorectal cancer tumor tissues.

	Wnt5a		
	S isoform high & L isoform low (*n* = 41)	L isoform high & S isoform low (*n* = 27)	*p* value
β-Catenin			0.020
High (*n* = 32), *n* (%)	24 (58.5)	8 (29.6)	
Low (*n* = 36), *n* (%)	17 (41.5)	19 (70.4)	

## Discussion

The non-canonical Wnt signaling pathway has been investigated and found to directly promote the invasiveness and malignant progression of various types of cancer [25]. The non-canonical Wnt5a ligand activates the non-canonical Wnt signaling pathway. However, whether Wnt5a is capable of influencing CRC tumorigenesis is still ambiguous. The present study established for the first time the ambiguous role of Wnt5a in CRC due to the encoding of distinct roles of Wnt5a mRNA isoforms. There are several lines of evidence that support this conclusion which are listed below. First, a relatively high expression level of the Wnt5a-S mRNA isoform and a low expression level of the Wnt5a-L mRNA isoform were detected in CRC cell lines and specimens. Second, knockdown of endogenous expression of the Wnt5a-S mRNA isoform in HCT116 cells drastically inhibited their growth ability; in contrast, targeting the Wnt5a-L mRNA isoform by anti-Wnt5a-L isoform oligonucleotides in HCT116 cells dramatically augmented the cell growth ability. Third, cell viability was significantly reduced in Wnt5a-L mRNA isoform-overexpressed of HCT116 cells. Fourth, knockdown of the Wnt5a-S mRNA isoform caused apoptosis induction through inducing FASLG expression and reducing TNFRSF11B expression. Fifth, reactivation of the Wnt5a-L mRNA isoform by treatment with 5-Aza enhanced induction of apoptosis by the siWnt5a-S isoform. Collectively, this study is the first to report the distinct roles of Wnt5a mRNA isoforms in CRC tumorigenesis, as evidenced by studies using cell lines, clinical samples, and TCGA CRC dataset analyses.

It was demonstrated that activation of β-catenin due to an *APC* gene mutation was linked to the initiation of colorectal tumorigenesis [26]. However, our previous study showed that the low mutation rate (~34%) of the *APC* gene was found in CRC patients of a Taiwanese population [27], which is close to the reported level in Asia [28]. These results indicated that another factor exists to regulate the expression of β-catenin of CRC patients with a low mutation rate of APC. In the present study, slight downregulation of β-catenin protein expression was detected in 5-Aza-treated HCT116 cells at 50 μM (Fig 7C, left panel), but substantial inhibition of β-catenin protein expression was found in siWnt5a-S isoform-transfected HCT116 cells treated with 50 μM 5-Aza (Fig 7C, right panel). These results were further confirmed using CRC specimens to show that high levels of β-catenin mRNA expression in tumor tissues might be due to high mRNA expression levels of the Wnt5a-S isoform and low mRNA expression level of the Wnt5a-L isoform. Taken together, these results suggest that reactivation of the Wnt5a-L mTNA isoform alone by treatment with 5-Aza is insufficient to reduce β-catenin mRNA expression. Simultaneous reactivation of the Wnt5a-L mRNA isoform and knockdown of the Wnt5a-S mRNA isoform can enhance Wnt5a-L isoform-mediated downregulation of β-catenin expression.

The abundance of mRNAs have been put forward as prognostic markers for various of cancers [29]. In the present study, we found that highly expressed of Wnt5a-S mRNA isoform was detected in CRC cell lines and tissues, in contrast, downregulated of Wnt5a-L mRNA isoform was detected in CRC cell lines and tissues. Although, the expression of Wnt5a protein isoforms were also detected by using commercial available of pan-Wnt5a antibody, it is not possible to detect the presence or absence of a translated protein from the short Wnt5a mRNA isoforms. Thus, the exact association between expression status of Wnt5a isoforms and CRC only can determine by analyzed Wnt5a mRNA isoforms. Collectively, monitoring the respective mRNA expression levels of Wnt5a-L isoform and Wnt5a-S isoform may be a useful cancer biomarker.

In conclusion, our report describes how the expressions and functional roles of the Wnt5a mRNA isoforms differ in CRC. The Wnt5a-L mRNA isoform can suppress cell proliferation and act as tumor suppressor in CRC cells, while the Wnt5a-S mRNA isoform can promote cell proliferation and plays an oncogenic role in CRC cells. Furthermore, the methylation-inactivated Wnt5a-L mRNA isoform can be reactivated by treatment with a demethylation agent, and knockdown of expression of the Wnt5a-S mRNA isoform can enhance reactivation of Wnt5a-L isoform-mediated downregulation of β-catenin expression, eventually weakening tumor progression of CRC. Thus, the different Wnt5a mRNA isoforms can be used as novel prognostic markers for CRC in the future.

## Supporting information

S1 Table Association of pan-Wnt-5a protein expression and clinical parameters in tumor tissues of colorectal cancer patients. (DOCX)

S2 Table Association of pan-Wnt-5a protein expression and clinical parameters in tumor tissues of colorectal cancer patients. (DOCX)

S1 Fig The pan-Wnt5a protein analysis was conducted on protein isolated from eight CRC cell lines and one non-cancerous human CRL-1459 colon cell line. (TIF)

S2 Fig Wnt5a-L isoform inhibits growth of CRC cancer cells. (A) MTT assay of Wnt5a-L isoform overexpreed in HCCT116 cells (B) Expression of Wnt5a-L isoform, Cyclin D1, CDk4, and PARP were detected in Wnt5a-L isoform-overexpressed HCT116 cells by Western blot.
(TIF)

S3 Fig Pan-Wnt5a protein expression by treatment with 5-azacytidine (5-Aza) at the indicated concentration. (TIF)

S1 File Data file of Fig 1. (XLSX)

S2 File Data file of Fig 2. (XLSX)

S3 File Data file of Fig 3. (XLSX)

S4 File Data file of Fig 4. (XLSX)

S5 File Data file of Fig 6. (XLSX)

S6 File Data file of Fig 7. (XLSX)
