# Supplementary material for: Distinct roles and differential expression levels of Wnt5a mRNA isoforms in colorectal cancer cells
Source: PLoS One. 2017 Aug 31;12(8):e0181034. doi: 10.1371/journal.pone.0181034 (PMC5578641; doi:10.1371/journal.pone.0181034)
Supplement: S1 Table — (DOCX) [file pone.0181034.s001.docx]

**S1 Table. Association of pan-Wnt-5a protein expression and clinical parameters in tumor tissues of colorectal cancer patients.**

**pan-Wnt-5a**

______________________________________________

**Parameters** Low High *p* value

(n=47) (n=76)

Age (years)

≦65 28 34

>65 19 42 0.138

Gender

Female 19 45

Male 28 31 0.063

T factor

1 2 2

2 14 11

3 16 39

4 15 24 0.137

T factor

1+2 16 13

3+4 31 63 0.031

N factor

0 23 28

1+2 24 48 0.194

M factor

0 28 62

1 19 14 0.011

TNM Stage

I 13 10

II 4 18

III 10 34

IV 20 14 0.001
