# Supplementary material for: Distinct roles and differential expression levels of Wnt5a mRNA isoforms in colorectal cancer cells
Source: PLoS One. 2017 Aug 31;12(8):e0181034. doi: 10.1371/journal.pone.0181034 (PMC5578641; doi:10.1371/journal.pone.0181034)
Supplement: S2 Table — (DOCX) [file pone.0181034.s002.docx]

**S2 Table. Colorectal cancer cell lines used in this study.**

| **Cell line** | **Cell type** | **Provider** |
| --- | --- | --- |
| CRL1459 | Normal colon cells | Prof. PJ Lu |
| DLD1 | Colorectal adenocarcinoma cells | Prof. YW Cheng/Prof. H Lee |
| HCT15  WiDr  HT29 | Colorectal adenocarcinoma cells  Colorectal adenocarcinoma cells  Colorectal adenocarcinoma cells | Prof. YW Cheng/Prof. H Lee  Prof. YW Cheng/Prof. H Lee  Prof. YW Cheng/Prof. H Lee |
| Lovo  SW480  HCT116^WT^  HCT116^p53-/-^ | Colorectal adenocarcinoma cells  Colorectal adenocarcinoma cells  Colorectal adenocarcinoma cells  Colorectal adenocarcinoma cells | Prof. YW Cheng/Prof. H Lee  Prof. YW Cheng/Prof. H Lee  Prof. YW Cheng/Prof. H Lee  Prof. YW Cheng/Prof. H Lee |
