# Supplementary figures and images for: Distinct roles and differential expression levels of Wnt5a mRNA isoforms in colorectal cancer cells
Source: PLoS One. 2017 Aug 31;12(8):e0181034. doi: 10.1371/journal.pone.0181034 (PMC5578641; doi:10.1371/journal.pone.0181034)

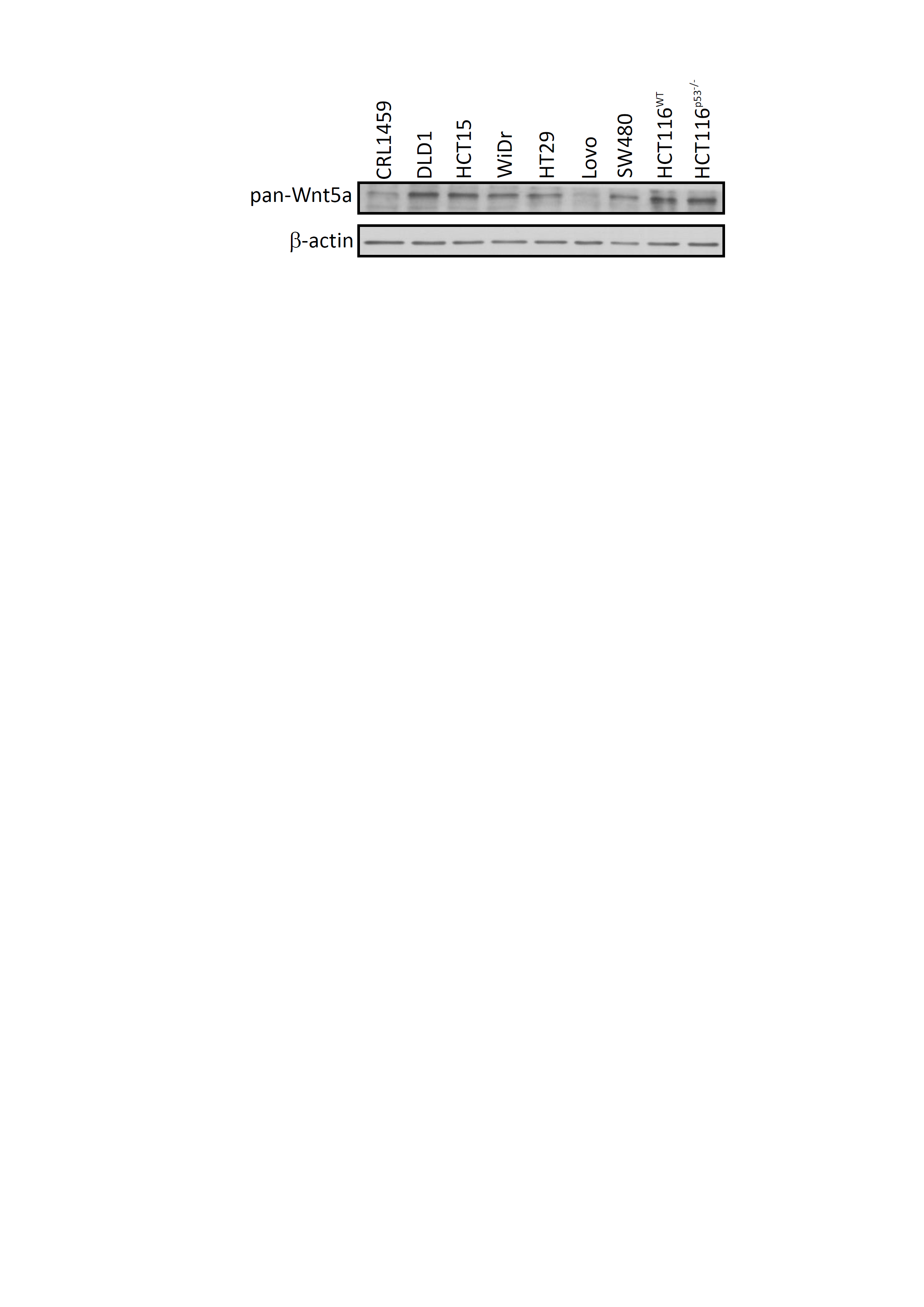

Supplement: S1 Fig — (TIF) [file pone.0181034.s003.tif]

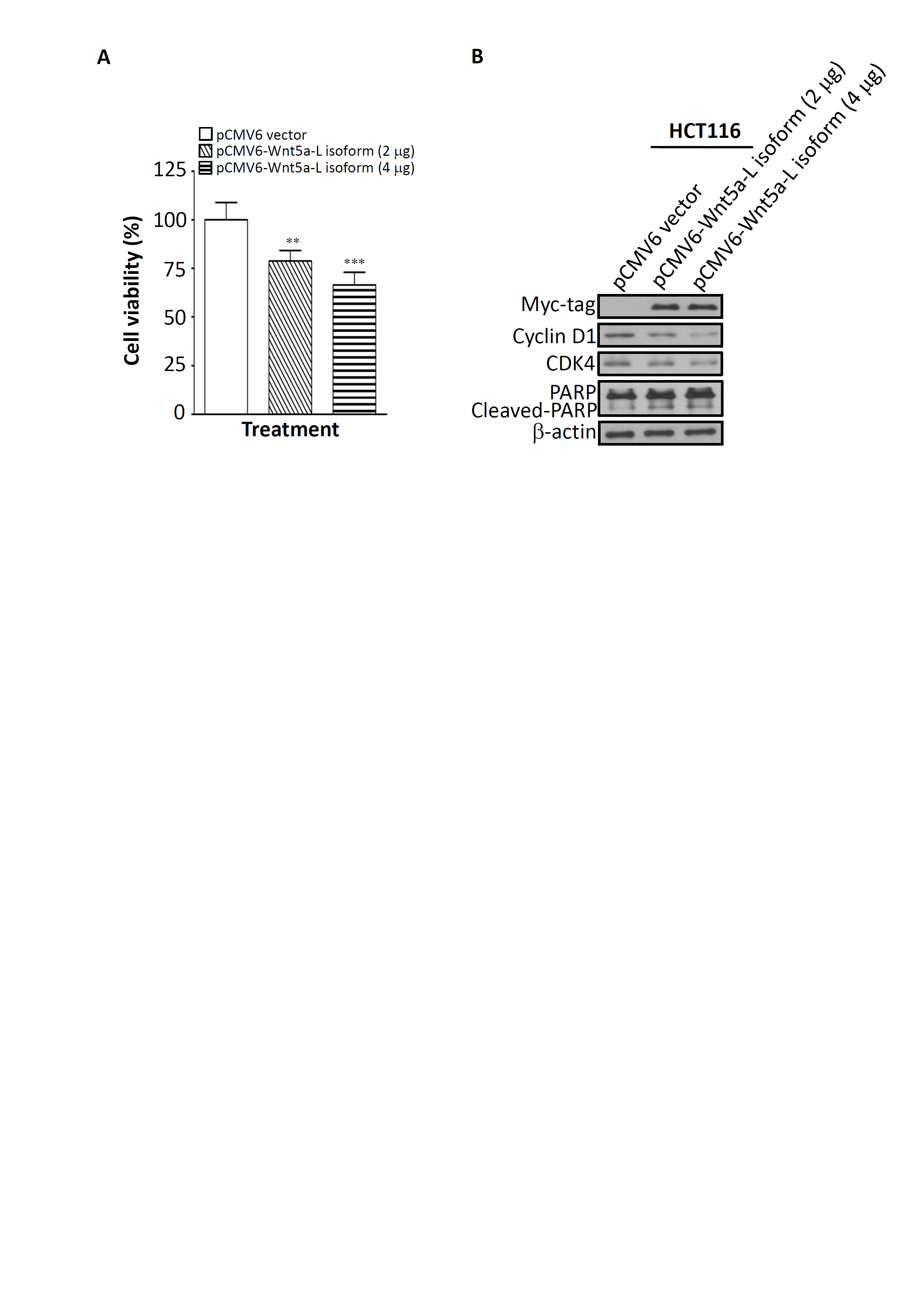

Supplement: S2 Fig — (A) MTT assay of Wnt5a-L isoform overexpreed in HCCT116 cells (B) Expression of Wnt5a-L isoform, Cyclin D1, CDk4, and PARP were detected in Wnt5a-L isoform-overexpressed HCT116 cells by Western blot. (TIF) [file pone.0181034.s004.tif]

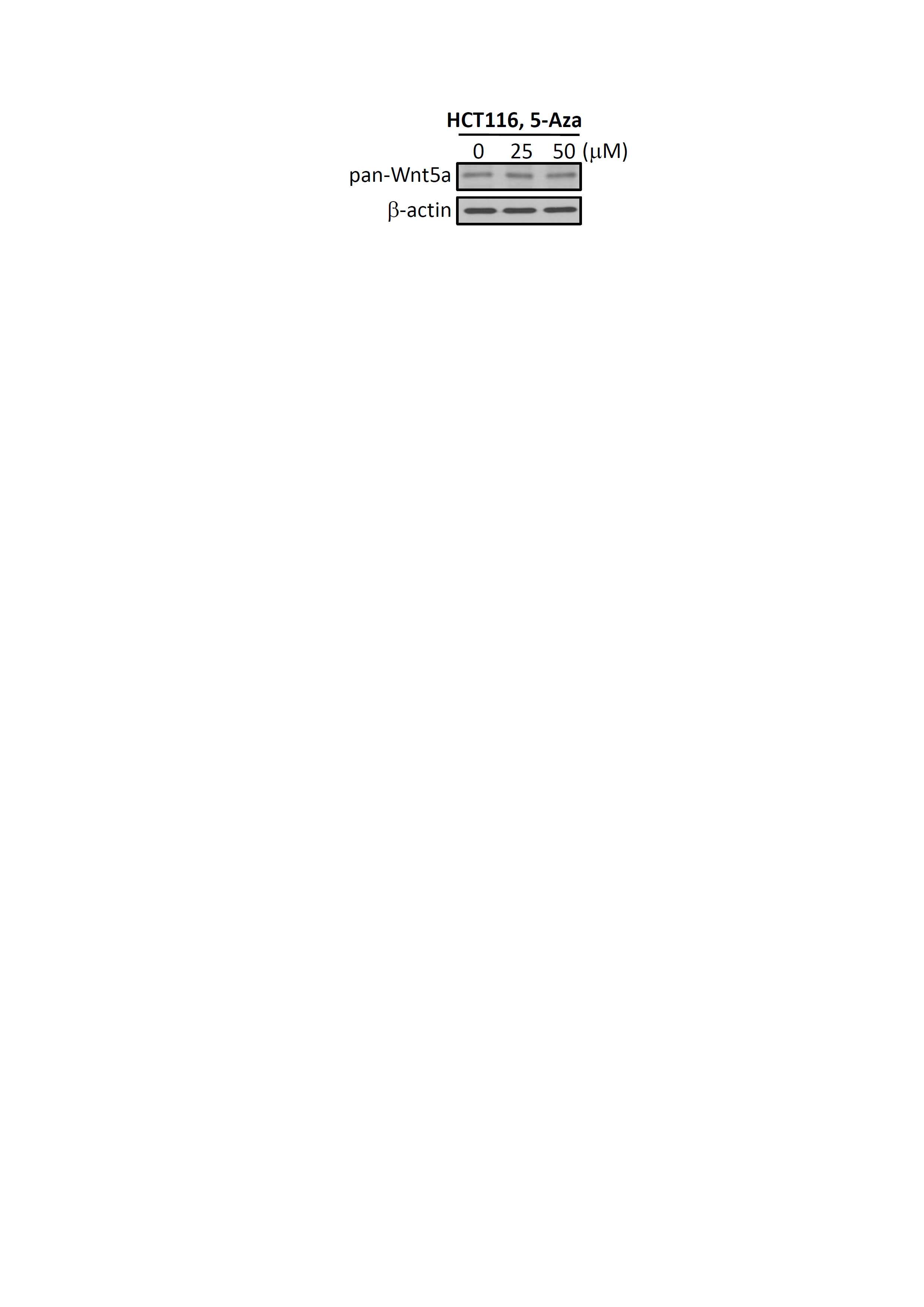

Supplement: S3 Fig — (TIF) [file pone.0181034.s005.tif]
